# Characterization of Photoluminescent Polylactone-Based Nanoparticles for Their Applications in Cardiovascular Diseases

**DOI:** 10.3389/fbioe.2019.00353

**Published:** 2019-11-22

**Authors:** Aneetta E. Kuriakose, Nikhil Pandey, Dingying Shan, Subhash Banerjee, Jian Yang, Kytai T. Nguyen

**Affiliations:** ^1^Bioengineering Department, The University of Texas at Arlington, Arlington, TX, United States; ^2^Department of Biomedical Engineering, Pennsylvania State University, University Park, PA, United States; ^3^Division of Cardiology, VA North Texas Medical Center, Dallas, TX, United States; ^4^Department of Internal Medicine, The University of Texas Southwestern Medical Center, Dallas, TX, United States

**Keywords:** BPLP, bioimaging, toxicity, vascular drug carriers, endothelial cells, cardiovascular disease, theranostics

## Abstract

Cardiovascular diseases (CVD) affect a large number of the population across the globe and are the leading cause of death worldwide. Nanotechnology-based drug delivery has currently offered novel therapeutic options to treat these diseases, yet combination of both diagnostic and therapeutic abilities is further needed to understand factors and/or mechanisms that affect the treatment in order to design better therapies to challenge CVD. Biodegradable photoluminescent polylactones (BPLPLs) enable to bridge this gap as these materials exhibit a stable, long-term intrinsic fluorescence as well as offers excellent cytocompatibility and biodegradability properties. Herein, we formulated three different BPLPL based nanoparticles (NPs), including BPLP-co-poly (L-lactic acid) (BPLPL-PLLA), BPLP-co-poly (lactic-co-glycolic acid) copolymers with lactic acid and glycolic acid ratios of 75:25 (BPLPL-PLGA75:25) and 50:50 (BPLPL-PLGA50:50), and extensively evaluated their suitability as theranostic nanocarriers for CVD applications. All BPLPL based NPs were <160 nm in size and had photoluminescence characteristics and tunable release kinetics of encapsulated protein model depending on polylactones copolymerized with BPLP materials. Compared to BPLPL-PLLA NPs, BPLPL-PLGA NPs demonstrated excellent stability in various formulations including deionized water, serum, saline, and simulated body fluid over 2 days. *In vitro* cell studies with human umbilical vein derived endothelial cells showed dose-dependent accumulation of BPLPL-based NPs, and BPLPL-PLGA NPs presented superior compatibility with endothelial cells in terms of viability with minimal effects on cellular functions such as nitric oxide production. Furthermore, all BPLPL NPs displayed hemocompatibility with no effect on whole blood kinetic profiles, were non-hemolytic, and consisted of comparable platelet responses such as platelet adhesion and activation to those of PLGA, an FDA approved material. Overall, our results demonstrated that BPLPL-PLGA based NPs have better physical and biological properties than BPLPL-PLLA; hence they have potential to be utilized as functional nanocarriers for therapy and diagnosis of CVD.

## Introduction

Stenting and balloon angioplasty are common endovascular strategies used to open the occluded blood vessel. However, such interventions often damage the arterial wall, allowing activation and binding of circulating platelets to the exposed subendothelium that initiate inflammatory responses, ultimately leading to the development of restenosis. Although emerging drug eluting technologies have reduced the rates of endovascular complications, their long-term efficacy is hindered by late thrombosis and the catch-up phenomenon of restenosis (Sun et al., 2015). It is now well-known that ineffective reconstitution of the endothelial layer often results in these late stage complications. To overcome these limitations, several strategies involve with stem cell therapies and drug carriers have been developed. This includes the administration of endothelial progenitor cells (EPCs) (Povsic and Goldschmidt-Clermont, 2008; Chen et al., 2016, 2017), biodegradable superparamagnetic nanoparticles loaded endothelial cells (ECs) (Polyak et al., 2016; Vosen et al., 2016), induced pluripotent stem cells-derived ECs (Adams et al., 2013; Giordano et al., 2016) and post angioplasty strategies to restore vascular integrity. Vascular targeted nanocarriers were also employed to deliver anti-inflammatory or anti-mitogenic agents such as paclitaxel, docetaxel, simvastatin, or everolimus to inhibit intimal hyperplasia and provide a suitable environment for endothelial regeneration (Chan et al., 2010). Previously, we have developed ~400nm sized polymeric nanoscaffolds that interface with the injured arterial endothelium via glycoprotein 1bα ligand and capture circulating EPCs via anti-CD34 antibodies (Su et al., 2014). These multifunctional nanosystems cloaked the denuded endothelium, prevented platelet-mediated reactions, and reduced subsequent neointimal formation. Furthermore, they promoted rapid endothelial reconstruction by locally capturing EPCs and supporting their adhesion. Despite these achievements, the therapeutic potential of the nanoscaffolds was not fully achieved as indicated by a decreased the binding of EPCs to the injured artery after 7 and 21 days of transplantation, delayed intimal hyperplasia formation, as well as incomplete endothelial regeneration process. This might be associated with ineffective margination and retention of EPCs or nanoscaffolds on the damaged vascular wall either due to hydrodynamic dislodging forces exerted on them by circulation or their poor tissue interactions.

The real time, non-invasive monitoring of transplanted cells and nanocarriers after delivery would help us to determine their pharmacokinetics and tissue distribution *in vivo*. In addition, we can estimate the required dosing of the therapeutic candidates to be administered at the injured site, assess the outcome of the therapy and develop more efficient treatment/ delivery strategies. A common strategy employed in labeling cells or drug carriers for imaging applications is by directly incorporating fluorophores, radioisotopes, quantum dots, and paramagnetic nanoparticles within them. However, major concerns involved with these imaging agents are often associated with their poor photobleaching-resistance and substantial cytotoxicity, which limit their applications for long-term *in vivo* tracking of cells and/or drug carriers. Another technique to image living cells involves genetic modification by introducing reporter genes into the cells' genome to express specific fluorescent/bioluminescent proteins or enzymes required for signal generation. This approach is less favorable as it produces gene alteration, and often requires viral vectors for gene transduction, which may cause immunogenicity and mutagenesis. Therefore, this strategy of imaging is only approved in terminally ill patients (Wang and Jokerst, 2016). Considering the issues associated with the tracking of cells and/or drug carriers using the aforementioned strategies, the development of biodegradable and biocompatible materials that allows non-invasive, stable and long-term imaging capabilities has become increasingly desirable.

Earlier, we have developed citrate-based biomaterials, known as biodegradable photoluminescent polymers (BPLPs) that possessed a strong and tunable photoluminescence phenomenon; and demonstrated their potential use in bioimaging, drug delivery and tissue engineering (Yang et al., 2009). Unlike other imaging agents that are not degradable, BPLPs are created from biocompatible monomers via a convenient thermal polycondensation reaction and shown to have controlled degradability properties. However, the main challenge of using BPLPs for nanoparticle fabrication was associated with their low molecular weight, which resulted in nanoparticle aggregation in physiological conditions, hence limiting their use as an imaging probe. To overcome this, we synthesized new polymers by incorporating BPLPs into the widely used biodegradable polylactones, referred to as biodegradable photoluminescent polylactones (BPLPLs) that showed higher molecular weight, improved mechanical strength, and favorable processability over BPLPs (Xie et al., 2014; Hu et al., 2016). The intrinsic and stable fluorescent property of BPLPs is well-preserved in BPLPLs. Furthermore, the BPLPLs fluorescence emission ranging from blue to red can be adjusted by varying different amino acids in the syntheses of BPLPs (Yang et al., 2009; Xie et al., 2017).

In this research, we developed three different nanoparticles based on BPLPLs including BPLP-co-poly (L-lactic acid) (BPLPL-PLLA) and BPLP-co-poly (lactic-co-glycolic acid) copolymers with lactic acid and glycolic acid ratios of 75:25 (BPLPL-PLGA75:25) as well as 50:50 (BPLPL-PLGA50:50). Furthermore, we have characterized for their physical properties and biocompatibility with the blood cells and endothelial cells and investigated for their bioimaging applications. Our preliminary characterization studies would help us to identify a suitable BPLPL-based material to synthesize theranostic NPs that can be utilized both as an imaging agent to track the EC delivery and as a vascular drug carrier to promote *in situ* reendothelialization post arterial injury.

## Experimental Procedures

### Materials

Synthesis of BPLPLs such as BPLPL-PLLA (1:100), BPLPL-PLGA50:50 (1:100), BPLPL-PLGA75:25 (1:100) was described previously (Xie et al., 2014; Hu et al., 2016). The ratio of 1:100 represents the feeding molar ratio of BPLP either with lactic acid or a combination of lactic acid and glycolic acid. PLGA50:50 of molecular weight 55–65 kDa was purchased from Akina, Inc. (West Lafayette, IN). Other reagents including bovine serum albumin (BSA) and polyvinyl alcohol (PVA) of molecular weight 31–50 kDa were brought from Sigma-Aldrich (St. Louis, MO). MTS reagent (CellTiter 96®AQueous One Solution Cell Proliferation Assay) and Pierce BCA protein assay were obtained from Promega (Madison, WI) and ThermoFisher Scientific (Grand Island, NY), respectively. OxiSelect™ Intracellular Nitric Oxide (NO) Fluorometric Assay Kits were purchased from CellBioLabs, Inc. (San Diego, CA). Furthermore, human umbilical vein endothelial cells (HUVECs) was purchased from American Type Culture Collection (ATCC, Manassas, VA), while the culture media (Vasculife Basal Medium) and supplemental kits (Vasculife VEGF Lifefactors) were purchased from Lifeline Cell Technology (Frederick, MD). Other chemicals, if not specified were purchased from Sigma Aldrich.

### Synthesis of BPLPL-Based Nanoparticles

BSA was selected as the model protein to be encapsulated into BPLPL nanoparticles, which were synthesized by a standard double emulsion technique. For this procedure, BSA solution (20 mg of BSA dissolved in 0.2 ml of DI water) was emulsified into 2% (w/v) BPLP-polylactones solution prepared in 5 mL of chloroform and sonicated. This primary emulsion was added drop wise into 12 mL of 5% (w/v) PVA and sonicated again at 30 W for 5 min. Following the overnight stirring to evaporate organic solvents, the nanoparticles were washed and isolated by centrifugation at 15,000 rpm for 30 min, and protein (BSA)-loaded BPLPL based NPs were collected via freeze-drying. Blank BPLPL NPs as well as PLGA50:50 NPs were also fabricated using similar procedures without adding BSA to be utilized for *in vitro* cells- and blood-based studies.

### Physical Characterization of BPLPL-Based Nanoparticles

The nanoparticles were characterized for their particle size, polydispersity and zeta potential via a dynamic light scattering (DLS) method using Zeta PALS zeta potential analyzer (Brookhaven Instruments, Holtsville, NY). The size and morphology of the nanoparticles were also observed using transmission electron microscopy (TEM). The stability of particles was determined by observing the variation in their size while suspended in various formulations such as DI water, saline (0.9% sodium chloride solution), 10% Fetal Bovine Serum (FBS, Atlanta Biological, Lawrenceville, GA), or simulated body fluid with similar composition of blood plasma, prepared as described previously Marques et al., 2011). The particles were incubated at 37°C and their sizes were measured using DLS every 12 h up to 3 days. Furthermore, the amount of BSA encapsulated into BPLPL-based particles was estimated based on unentrapped BSA in PVA solution after centrifugation. The percentage of loading efficiency was calculated as actual amount of BSA loaded with respect to the initial amount of BSA used to prepare NPs. For the *in vitro* release study of BSA, 1 mg/ml of particle solution in PBS suspended in a 100 kDa dialysis bag (Spectrum Laboratories Inc., Rancho Dominguez, CA) was dialyzed against phosphate buffer saline (PBS) solution. At each predetermined time points, 1 ml of dialysate solution was collected and replaced with fresh PBS solution. BSA content in the collected solution was quantified using BCA protein assays following manufacturer's instructions, and cumulative BSA release over the time was analyzed based on BSA standards. *In vitro* degradation of nanoparticles in DI water was analyzed over a period of 4 weeks. Briefly, NPs was suspended in DI water and incubated at 37°C for predetermined times. At each time point, particles were collected and freeze dried. The degradation was determined based on the remaining mass of NPs.

### *In vitro* Cell Studies of BPLPL-Based Nanoparticles

#### Cytocompatibility

To evaluate the cytotoxicity of BPLPL-based nanoparticles with human umbilical vein endothelial cells (HUVEC), cells were seeded in 96 well plates at a seeding density of 30,000 cells/cm^2^ and incubated in 37°C for 24 h. Following incubation, cell culture media was replaced with increasing concentrations of nanoparticle suspension (in media) for 24 h. The cells were then washed and incubated with MTS assay reagents for 3 h. Absorbance readings was measured at 490 nm using UV-Vis spectrophotometer (Infinite M200 plate reader, Tecan, Durham, NC), and the percent of cell viability was determined with respect to untreated cells.

#### Cellular Uptake

The efficiency of HUVECs to internalize BPLPL-based nanoparticles was determined. Briefly, HUVECs of density 30,000 cells/cm^2^ were initially seeded in to 96 well plates and allowed to attach for 24 h. The cell culture media was then replaced with nanoparticle suspensions of various concentrations, and the plates were incubated for 4 h. After treatment, cells were washed with PBS and lysed with 1% Triton X-100 for 30 min at 37°C, and lysate was utilized to measure the nanoparticles' fluorescence intensities at excitation and emission wavelength of 377 and 431 nm, respectively. These measurements were analyzed against a nanoparticle standard. These fluorescence intensity values were then normalized with the total protein content per sample using BCA assays following manufacturer's instructions. In parallel, the nanoparticle interactions with endothelial cells were imaged using a fluorescence microscope under FITC channel.

#### Cellular Functionality

HUVEC functionality in the presence of BPLPL-based nanoparticles was determined based on nitric oxide (NO) production. Nanoparticles (1 mg/ml) were incubated with cells for 24 h, following which nitric oxide production of exposed cells was quantified using Intracellular Nitric Oxide Fluorometric Assay kits (Cell Biolabs, Inc., San Diego, CA) following the manufacturer's instructions. In brief, a NO fluorometric probe (provided with the kit) enters the cells and deacetylates by intracellular esterase to a non-fluorescent intermediate, which is rapidly oxidized by nitric oxide into a fluorescent triazolo-fluorescein analog. The fluorescence intensity is proportional to NO levels within the cell cytosol, which can be quantified at a wavelength of 480 nm (excitation)/530 nm (emission) using UV/vis spectrophotometer (Infinite M200 plate reader, Tecan, Durham, NC). Cells grown on tissue culture plates without any treatment served as control.

### *In vitro* Blood Studies of BPLPL-Based Nanoparticles

#### Blood Collection

Whole blood was drawn from healthy adult volunteers into acid citrate dextrose anticoagulant tubes (ACD, Solution A; BD Franklin Lakes, NJ). Consent from the volunteers was obtained prior to the blood collection, and all the procedures strictly adhered to the IRB standards approved at the University of Texas at Arlington.

#### Whole Blood Clotting Kinetics and Hemolysis

Briefly, in hemolysis, 10 μL of various concentrations of nanoparticles ranging from 0 to 1,000 μg/ml were incubated with 200 μl of blood for 2 h at 37°C. The nanoparticles were centrifuged at 1,000 rpm for 5 min and absorbance of the supernatant was obtained using UV-Vis Spectrophotometer at a wavelength of 545 nm. Blood diluted in DI water served as the positive control, whereas saline diluted blood as the negative control for hemolysis studies. Percentage of hemolysis due to each sample was quantified based on Equation (1). In the whole blood clotting study, we studied the effects of particles on normal blood clotting kinetics, which was measured as blood clotting index (BCI). Here, whole blood initially activated by adding 0.01 M of calcium chloride, and 50 μL of activated blood was then treated with 10 μL of 0.5 mg/ml of nanoparticles for predetermined time points. At each time point, 1.5 ml of DI water was added to lyse the un-clotted blood, and absorbance of supernatant was measured at 540 nm. Untreated blood served as a control. The absorbance of whole blood (without any addition of calcium chloride) in water at 540 nm was applied as a reference value. The BCI can be quantified from Equation (2).

(1) $$\begin{array}{r} Hemolysis\;\left(\%\right)=\;\frac{Absorbance\;of\;sample-Absorbance\;of\;negative\;control}{Absorbance\;of\;positive\;control-Absorbance\;of\;negative\;control}X\;100\% \end{array}$$

(2) $$\begin{array}{r} BCI=\;\frac{Absorbance\;of\;blood\;in\;contact\;with\;samples\;at\;set\;time\;points\;at\;545nm}{Absorbance\;of\;whole\;blood\;in\;water\;at\;545nm\;at\;time\;0} \end{array}$$

#### Platelet Adhesion and Aggregation

We further investigated the hemocompatibility of BPLPL materials based on the platelet adhesion and activation. For this study, platelet rich plasma (PRP) was collected by centrifuging whole blood at 190 g for 12 min. PRP was incubated with BPLPL films for 1 h at 37°C under static conditions. After 1 h, films were rinsed carefully with PBS and attached platelets were lysed using cell lysis solution for 1 h. The LDH release corresponding to platelet adhesion was quantified by detecting the amount of lactate dehydrogenase (LDH) present in the lysate solution using CytoTox 96^®^ Non-Radioactive Cytotoxicity Assays according to the manufacturer's instructions. Glass served as the positive control for comparison. The morphology of platelet adhesion on the polymer films were also visualized using scanning electron microscopy (SEM) imaging. Briefly, platelets on films were fixed with 2.5% glutaraldehyde (Electron Microscopy Science, 16536-15) for overnight, post fixed with 1% Osmium tetroxide in 0.1 M Cacodylate buffer (Electron Microscopy Sciences, 19150) for 1 h, dehydrated with graded series of ethanol (50, 75, 95, 100%) for 15 min at each step, and further dried using varying ratios of hexamethyldisilane (HMDS) in ethanol (1:2, 1:1, 2:1) for 15 min at each step. Finally, the films were dried using 100% HMDS for 30 min and then sputter-coated with the silver for SEM. In addition, after incubating PRP with polymer films for 1 h, 5 μL of suspension was collected and incubated with saturating concentrations of CD42b-PE (platelet marker) and PAC1- FITC (activated glycoprotein GP IIb/IIIa receptor marker) for 20 min. The antibodies were obtained from BD Biosciences. The platelets were fixed with 1% paraformaldehyde for 2 h in 4°C and were analyzed on BD LSRII flow cytometer. At least 10,000 events per sample were analyzed and identified based on their forward and side scattering characteristics and by positive staining with anti-CD42b-PE antibodies. The percentage of GpIIb/IIIa expressing platelets was calculated relative to the total number of platelets (CD42b positive cells).

### Statistical Analysis

All the experiments were performed with *n* = 3–6 if not specified. Data were expressed as mean ± SEM. The statistical analysis was assessed using ANOVA followed by *post-hoc* Pairwise Multiple Comparisons using Holm–Sidak method on GraphPad Prism (GraphPad Software Inc., CA). A significant difference was considered where *P*-values appeared < 0.05.

## Results

### Physical Characterization of BPLPL-Based NPs

DLS results show that the BPLPL-PLGA50:50, BPLPL-PLGA75:25 and BPLPL-PLLA based nanoparticles suspended in DI water have hydrodynamic sizes of 157, 149, and 145 nm, respectively (Table 1).

**Table 1:** Size, charge, polydispersity of BPLPL based NPs.

**Polymeric Nanoparticles**	**Size (nm)**	**PD**	**Zeta potential (mV)**
BPLPL-PLLA NPs	145 ± 26	0.129 ± 0.035	−21.85 ± 2.16
BPLPL PLGA75:25 NPs	149 ± 37	0.116 ± 0.032	−23.85 ± 1.32
BPLPL-PLGA50:50 NPs	157 ± 44	0.164 ± 0.010	−24.72 ± 0.82
PLGA 50:50 NPs	176 ± 36	0.130 ± 0.032	−15.46 ± 1.52

**Figure 1:**
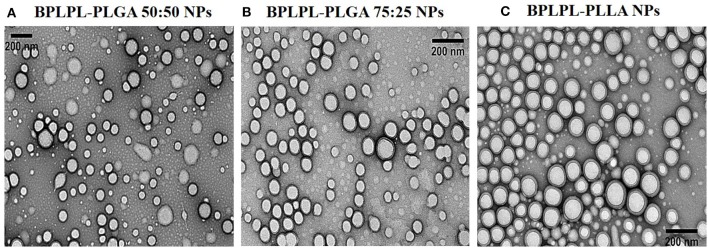
Morphological characterization of BPLPL-based NPs. TEM images of **(A)** BPLPL-PLGA50:50 (1:100), **(B)** BPLPL-PLGA75:25(1:100), **(C)** BPLPL-PLLA (1:100) shows uniform sized and spherical morphology of nanoparticles. The scale bar represents 200 nm.

**Figure 2:**
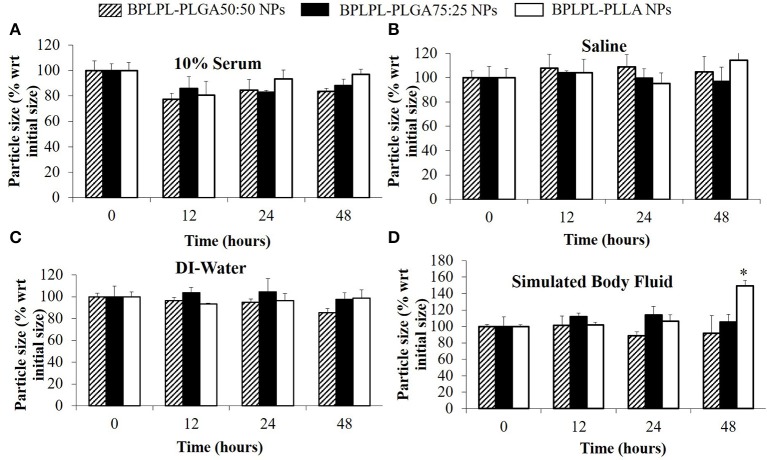
Stability of BPLPL-based NPs. NP stability in various formulations such as **(A)** 10% serum, **(B)** Saline (0.9% NaCl), **(C)** DI-water, and **(D)** Stimulated body fluid based on particle size measured over periods of 48 h. Graph plot as average ± SD of *n* = 3 samples. Asterisk (*) represents *p* < 0.05 in comparison to size of BPLPL-PLLA NPs at initial time point.

The polydispersity values ranging from 0.10 to 0.17 suggest the particles were well-dispersed. TEM images of BPLPL nanoparticle also confirms the uniform distribution of NPs with smooth and spherical morphology (Figures 1A–C). Zeta potential for BPLPL-PLGA 50:50, BPLPL-PLGA 75:25 and BPLPL-PLLA based nanoparticles were −24.7, −23.9, and −21.9 mV, respectively, suggesting that the particles might be stable in physiological solutions (Honary and Zahir, 2013). In addition, the stability of BPLPL based nanoparticles was evaluated at various formulations including DI water, 10% FBS, 0.9% saline and simulated body fluid by recording the nanoparticles diameter at fixed time intervals. We have observed that BPLPL-PLGA NPs were stable in all formulations for 48 h with no significant aggregation or change in size. Although BPLPL-PLLA NPs remained relatively stable in DI water, saline and serum, they tended to have some aggregation in simulated body fluid at 48 h (Figure 2).

**Figure 3:**
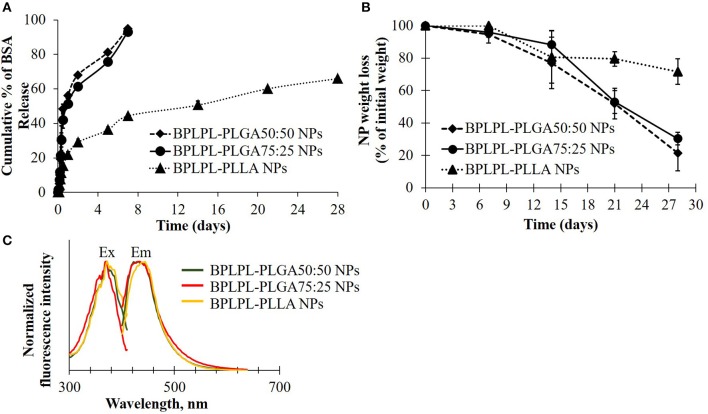
Physical characterization of BPLPL-based NPs. **(A)** BSA release kinetics from BPLPL-based nanoparticles in PBS solution at 37°C for 2 weeks, **(B)** Degradation of NPs in water at 37°C for 4 weeks, and **(C)** Fluorescence spectrum of cysteine derived BPLPL-based NPs (1 mg/ml) has an excitation and emission at 377 and 441 nm, respectively. Graph plot as average ± SD of *n* = 3 samples.

To determine if these nanoparticles could be utilized for drug delivery applications, their drug release kinetics and degradation studies were conducted. BSA was chosen as the model growth factor. BPLPL-PLGA 50:50, BPLPL-PLGA 75:25 and BPLPL-PLLA showed a loading efficiency of 70, 69, and 77%, respectively. Figure 3A showed that both BPLPL-PLGA nanoparticles could release ~50% content within 24 h, and the complete release was achieved in 7 days. On the other hand, BPLPL-PLLA nanoparticles demonstrated comparatively lower release kinetics, and only 50% BSA release was achieved in 2 weeks. The *in vitro* degradation study also emphasizes the role of polymer composition on particle behavior (Figure 3B), where BPLPL-PLGA particles showed a similar degradation rate and almost ~80% degraded in 4 weeks. However, BPLPL-PLLA degraded slowly with only ~30% lost in weight when observed after 4 weeks. Furthermore, all BPLP-cys-polylactone based nanoparticles showed maximum excitation and emission wavelength at 374 and 441 nm, respectively (Figure 3C).

### *In vitro* Cell Studies With BPLPL-Based Nanoparticles

**Figure 4:**
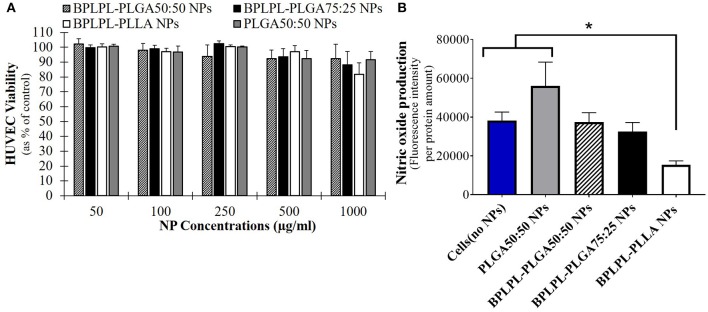
Cytocompatibility of BPLPL-based nanoparticles. **(A)** HUVEC viability in presence of various concentrations of nanoparticles as quantified using MTS assays; **(B)** Intracellular NOS activity within HUVECs was quantified after incubation with 1,000 μg/ml of BPLPL-based NPs for 24 h. Fluorescence intensity correlates with NOS activity or NO production within cells. *Represents significance with respect to cells treated to BPLPL-PLLA. Graph plotted in terms of average ± standard error mean (SEM).

Cytocompatibility evaluation of BPLPL-based NPs at various concentrations after 24 h incubation with HUVECs was conducted. Accordingly, BPLPL NPs at all concentrations ranging from 50 to 1,000 μg/ml was shown to be compatible with HUVECs with >80% viable cells after NP exposure (Figure 4A). In addition, the functional status of endothelial cells in the presence of BPLPL-based NPs was studied by assessing the nitric oxide (NO) production. Accordingly, we noted that NO production by HUVECs treated with BPLPL-PLLA NPs at 1,000 μg/ml for 24 h was significantly lower than that of cells treated with BPLPL-PLGA NPs and untreated cells (Figure 4B). On the other hand, NOS activity quantified for HUVECs in the presence of both BPLPL-PLGA50:50 and BPLPL-PLGA75:25 demonstrated no negative effects on cellular function.

**Figure 5:**
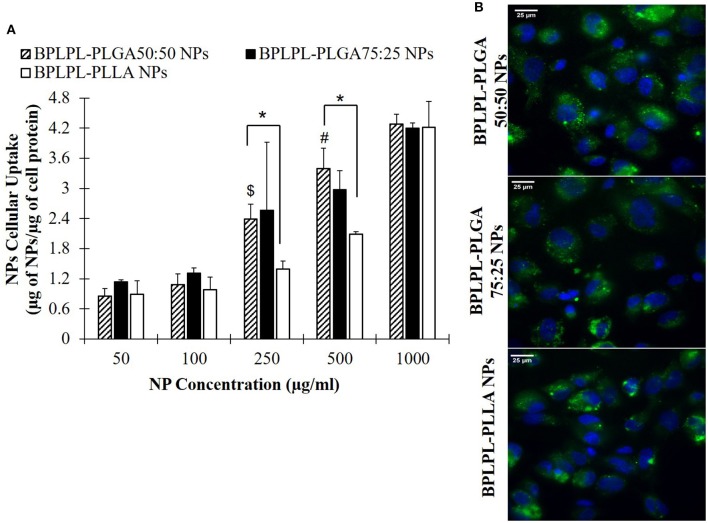
Cellular uptake of BPLPL-based nanoparticles. **(A)** Nanoparticle uptake by endothelial cells (HUVECs) at various concentrations after incubation for 4 hr in 37°C was quantified in terms of amount of nanoparticles relative to protein amount per sample. $ and # represents significance with respect to BPLPL-PLGA50:50 NPs at concentrations of 100 and 250 μg/ml, respectively; whereas * represents significance between BPLPL-PLGA50:50 NPs and BPLPL-PLLA NPs. **(B)** Fluorescent images of nanoparticles internalized HUVECs at 60X, green represents nanoparticles and blue for cell nuceli. The scale bar is 25 μm in length. Graph plotted in terms of average ± SEM.

Lastly, the uptake of BPLPL-based NPs by vascular endothelial cells (HUVECs) was studied by incubating cells with various concentrations of NPs over 4 h. All NP formulations showed dose-dependent cellular uptake up to a concentration of 1,000 μg/ml (Figure 5A). Fluorescence images also showed the internalization of BPLPL-based nanoparticles by endothelial cells and their subsequent localization in cytoplasmic region of cells after 4-h incubation with particles (Figure 5B).

### Hemocompatibility of BPLPL-Based Nanoparticles

**Figure 6:**
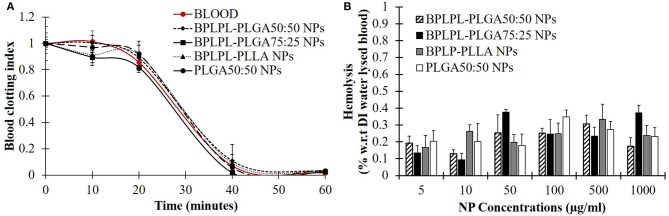
Hemocompatibility of BPLPL-based NPs. NP compatibility in whole blood was assessed based on **(A)** clotting kinetic profile in presence of 1 mg/ml of NPs and **(B)** hemolysis after incubating with nanoparticles of various concentrations for predetermined timepoints. Graph plot as average ± SEM.

**Figure 7:**
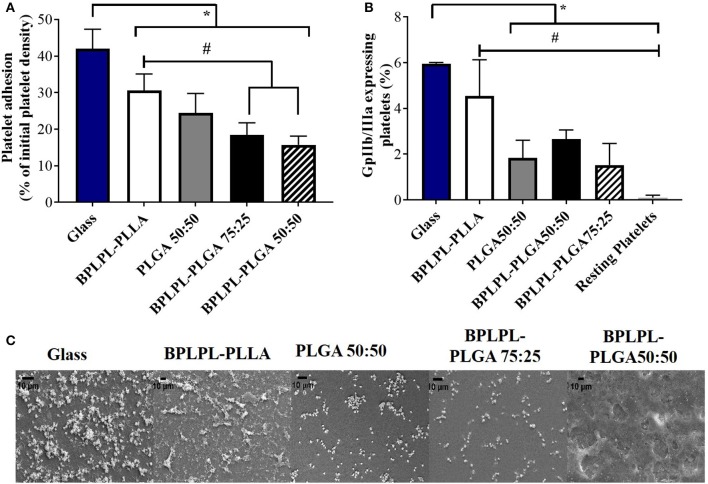
Platelet responses to BPLPL-based materials. **(A)** Platelet adhesion to BPLPL-based materials after 1-h incubation at 37°C quantified using LDH assays, **(B)** Platelet activation quantified based on expression of GPIIb/IIIa surface markers using flow cytometry, and **(C)** SEM images to demonstrate platelet morphological changes and interactions with BPLPL-based materials. *and # represents significance value of *p* < 0.05 with respect to glass and BPLPL-PLLA, respectively. Graph plot as average ± SEM, and scale bar for SEM images represents length of 10 μm.

Hemocompatibility of nanoparticles was determined based on whole blood clotting kinetics, hemolysis study, and platelet responses. Blood clotting time reflects the thromboresistance property of nanoparticles, and high thromboresistance (Blood clot index, BCI value) means high blood compatibility. All BPLPL-based nanoparticles showed similar BCI values with each other and when compared to untreated blood samples (Figure 6A). This suggests that particles have no significant effect on the normal blood clotting kinetics. Furthermore, BPLPL-based NPs at all tested concentrations proved to be non-hemolytic with a maximum of 0.4% which was well within the standardized ISO values for non-hemolytic materials, which is 0–2% (Cerda-Cristerna et al., 2011) (Figure 6B). To assess the compatibility of BPLPL-based materials with platelets, BPLPL films were incubated with PRP at 37°C for 1 h. Based on the analysis, BPLPL-PLLA showed a significantly higher number of platelets adhered onto its surface than those of BPLPL-PLGA counterparts (Figure 7A). As platelets get activated, P-selectin translocate from intracellular granules to the external membrane, whereas fibrinogen aggregates platelets by bridging glycoprotein GPIIb/IIIa between adjacent platelets (Merten and Thiagarajan, 2000). Based on flow cytometric analysis, significant amount of platelet activation was seen on the glass surface as a positive control (Figure 7B). The amount of activation seen on BPLPL-PLGA surface was similar to those on PLGA surfaces. In concordance to these observations, SEM images also showed significantly higher platelet attachment on glass and BPLPL-PLLA surfaces than others. Closer observation of these images (Figure 7C) shows that platelets are spreading and aggregating on glass and BPLPL-PLLA surfaces. Other surfaces such as PLGA 50:50, BPLPL-PLGA 75:25, and BPLPL-PLGA 50:50 also presented a platelet shape change representing the early stage of platelet activation, which was characterized by transformation from discoid to spheroid form with small bulbous protrusions distributed over the platelet surface (Zilla et al., 1987).

## Discussion

Bioimaging holds huge potential in the field of drug delivery, tissue engineering and regenerative medicine. During the last decade, several functional polymers with imaging capabilities were investigated to understand the biological processes and their potential applications for image-guided surgery and therapy (Braeken et al., 2017; Zhang and Yao, 2018). The BPLP is one such novel biomaterial with intrinsic and excellent photoluminescent properties that can be used as a label-free *in vivo* imaging tool for disease detection and treatment. The components that are utilized to develop BPLPs including citric acid, amino acids, and aliphatic diols, which are all commonly used in many FDA-regulated devices (Yang et al., 2009; Li et al., 2017). Recently, a family of BPLPLs based on BPLP have been reported (Xie et al., 2014). This new class of materials can be utilized to fabricate theranostic nanoparticles which can be tracked with a variety of microscopy techniques, including fluorescent microscopy, confocal laser scanning microscopy and two-photon microscopy (Li et al., 2017).

In our present work, we screened three different BPLPL-based nanoparticles (BPLPL-NPs) including BPLPL-PLLA, BPLPL-PLGA50:50, and BPLPL-PLGA75:25 to determine the most promising formulation that can be utilized for theranostic applications in treating cardiovascular diseases. Our characterization showed that most BPLPL-NPs maintained uniform, spherical morphology with 150 nm in diameter and higher negative zeta potential values compared to PLGA 50:50 nanoparticles due to inclusion of citric acid component in BPLP. This increased surface charged groups on BPLPL-based NPs not only provide additional functional motifs required for the conjugation of targeting ligands but also improve NPs stability in physiological fluids. To evaluate this, the diameter of BPLPL-NPs in various formulations including DI water, serum, saline, and simulated body fluid was monitored for 48 h. Accordingly, we found that most of BPLPL-NPs, except BPLPL-PLLA at 48 h in simulated body fluids were relatively stable with no signs of aggregations. It is plausible that with long-term incubation of BPLPL-PLLA in simulated body fluids alters the colloidal stability of particles due to enhanced interactions of these NPs with various salts and enzymes that constitute the solvent. Lazzari et al. (2012) have observed similar aggregation behavior for PLLA NPs in simulated body fluids after prolonged incubation. In such instances, simple modifications of BPLPL-PLLA NPs surface with PEG chains might be able to prevent the docking of enzymes or ions on nanoparticle surfaces, thereby improving their colloidal stability in physiological conditions (Singh et al., 2017).

The presence of PLLA in BPLPs also seem to affect the protein loading efficiency, protein release kinetics and degradation profile of BPLPL-PLLA NPs. It was noted that BPLPL-PLGA NPs released ~50% of BSA within 24 h, whereas BPLPL-PLLA achieved a similar amount of BSA release within 2 weeks. In addition, BPLPL-PLLA NPs demonstrated a slower degradation rate with ~30% lost weight compared to BPLPL-PLGA NPs which showed ~80% degradation within 4 weeks. Hu et al. (2016) explained that BPLP incorporation into polylactones could enhance water permeability, which in turn, accelerate the drug release rate and degradation of copolymers. Also, the percentage of hydrophilic glycolic acid in BPLPL would also attribute to faster degradation and thereby drug release from the particles. We speculate that the hydrophobic nature of PLLA, may have improved their protein encapsulation compared to its PLGA counterparts by forming a hydrophobic wall to retard BSA leakage into the outer water phase during the NP synthesis (Liu et al., 2006). Furthermore, the photoluminescent property of BPLP was retained in BPLPL-based NPs, with maximum excitation and emission wavelength at 344 and 441 nm, respectively. Previously, we have shown that depending on the amino acids used in BPLP syntheses, the fluorescence emission could been broadened up to 725 nm, highlighting the versatility of these polymers for biomedical imaging (Tran et al., 2009).

Following the physical and chemical characterization of BPLPL NPs, their cytotoxicity with endothelial cells was investigated. BPLPL NPs exhibited excellent cytocompatibility with >80% of HUVECs viability post treatment with NPs at all concentrations. Xie et al. (2014) also reported similar values for cell viability using 3T3 fibroblasts exposed to BPLPL-PLLA nanoparticles at concentrations ranging from 1 to 500 μg/ml. Due to the presence of high number of carboxylic groups on the BPLP backbone, it was noted earlier about the reduced cell survival in presence of BPLP NPs (Zhang et al., 2013; Xie et al., 2014). In our study, BPLPL-based NPs showed similar cytocompatibility profile as seen for PLGA NPs with minimal cytotoxicity on HUVECs. Similar to our observation, Hu et al. (2016) also reported comparable *in vitro* cytotoxicity by mesenchymal stem cells as well as *in vivo* foreign body response toward BPLPL and PLGA materials. This suggests that inclusion of BPLP into commonly used polymers such as PLGA did not significantly affect the cell survival while the newly synthesized polymers can still inherit the florescent properties from BPLP, which could be possibly utilized for theranostic applications in CVD treatment.

Next, the proper functioning of endothelial cells in presence of BPLPL-based NPs in terms of nitric oxide production was evaluated. Nitric oxide (NO) is an important signaling molecule released by endothelial cells to regulate vascular inflammation, platelet function, angiogenesis and protection from ischemia reperfusion injury. Any dysregulation of NO production due to NOS uncoupling is known to cause cardiovascular diseases (e.g., atherosclerosis, diabetes, and hypertension) (Le et al., 2017). Inorganic nanoparticles including fluorescent silica NPs, superparamagnetic iron oxide NPs, titanium dioxide NPs generally investigated to be utilized for bioimaging applications demonstrated to induce EC toxicity and dysfunction with impaired NO production (Montiel-Dávalos et al., 2012; Astanina et al., 2014; Cao, 2018). When compared to these NP types, BPLPL-PLGA NPs could be a better alternative since it demonstrated to be inert with no effect on normal cell activities. However, this is not the case with BPLPL-PLLA NPs, nitric oxide production by endothelial cells was significantly reduced when compared to cells exposed to PLGA NPs. The mechanism that influenced the cellular behavior in presence of BPLPL-PLLA NPs is not clear. However, it is plausible that the BPLPL-PLLA nanoparticles may have upregulated oxidative stress within the cells that can activate autophagy and eventually lead to endothelial dysfunction via the PI3K/Akt/mTOR pathway as seen for silica NPs (Duan et al., 2014). Similar to our observation, Wang et al. (2014) also noted that exposure of PLLA particles to human coronary artery endothelial cells decreased their NO production, and induce inflammatory adhesion molecule expression such as ICAM-1 and VCAM-1, which might facilitate immune cell adhesion and recruitment. Furthermore, several studies reported that stents coated with PLLA impairs endothelial cell functions and impaired their recovery on the luminal side of stents that promoted in stent late thrombosis (Liu and Ma, 2010; Xu et al., 2011).

To investigate the utilization of BPLPL-based NPs as an imaging probe to track HUVECs, we incubated BPLPL-based NPs with vascular endothelial cells (HUVECs) over time. Dose-dependent uptake of NPs was observed for all NP formulations. Fluorescence images demonstrated the internalization of BPLPL-based nanoparticles by endothelial cells and their subsequent localization in the cytoplasmic region of cells after a 4-h incubation. Our results agreed with previous reports from other groups that tested various nanoparticle formulations on a different cell line. For instance, Menon et al. (2014) demonstrated increasing uptake of PLGA nanoparticles by Type I alveolar epithelial cells up to 1,000 μg/ml. Kona et al. (2012) also observed dose-dependent uptake of GpIbα conjugated PLGA nanoparticles and unconjugated nanoparticles by human aortic endothelial cells, which were saturated at 300 μg/ml. Even BPLP particles were shown to have dose-dependent uptake characteristics (Wadajkar et al., 2012). In this study, they observed BPLP particles being uptake by the human dermal fibroblast without any saturation up to 500 μg/ml. It was also noted that hydrophilic and hydrophobic versions of BPLP polymers impacted the amount of nanoparticles being internalized by the different cell lines, thereby exhibiting variation in NP cellular uptake. Similarly, we have observed a significant difference in BPLPL-PLLA and BPLPL-PLGA50:50 based NPs uptake by endothelial cells, especially at concentrations of 250 and 500 μg/ml. We speculate that such difference is due to differential composition of PLLA and PLGA50:50 in BPLPLs materials, where PLLA more hydrophobic in nature than PLGA50:50 and thereby affected the NP uptake by the endothelial cells. Cells continued to exhibit significant uptake of these nanoparticles at 1,000 μg/ml, and as a result, almost similar amounts of NPs in the cells were observed at high concentrations despite of polymer types. On other hand, we did not observe cells demonstrate any dose-dependent NP uptake for BPLPL-PLLA NPs at concentrations <250 μg/ml. In line with our results, It is plausible that the serum proteins in the media interact with the nanoparticles and modulate their uptake kinetics by the endothelial cells at low concentrations (Lesniak et al., 2010; Pelaz et al., 2015). Also, at these small concentrations, the measured levels of fluorescence intensity produced by NPs in the cells may be more difficult to discriminate.

In addition to the intrinsic fluorescence property of BPLPLs, we investigated whether the citric acid composition endows them with hemocompatibility suitable for blood contacting applications as previously seen for poly(diol-citrate) (POC) prepared from citric acid and 1,8-octanediol (Yang et al., 2004; Motlagh et al., 2007; Tran et al., 2009, 2015). First, *in vitro* hemostatic properties of BPLPL-based NPs were evaluated by whole blood clotting experiment. At various time points, absorbance of RBCs that were not trapped in clots were determined at 540 nm. Higher BCI values represent reduced blood clotting kinetics, and we have observed blood treated with BPLPL-based NPs did not exhibit a different rate of clotting when compared either with untreated blood or blood incubated with PLGA NPs. Second, hemolytic results of BPLPL-based NPs demonstrate them to be non-hemolytic material that is safe to be utilized for drug delivery applications without causing any adverse effects. Lastly, platelet behaviors toward BPLPL-based materials indicates that BPLPL-PLGA consisted of better or comparable platelet attachment and activation as seen in those of PLGA surfaces; whereas, higher number of platelets adhered and expressed GPIIb/IIIa markers on BPLPL-PLLA surfaces. Many studies previously observed that PLLA in its unmodified form or without incorporation of therapeutic agents induce increased inflammatory responses mainly due to its hydrophobic nature (Nguyen et al., 2003; Meng et al., 2004; Okamura et al., 2011; Rudolph et al., 2015). Our speculation is that since incorporating BPLPs into PLLA shown to increase the wettability of the polymer (Hu et al., 2016); the effect we have observed for platelets to BPLPL-PLLA could be minimal than its unmodified form of PLLA, which must be investigated further.

## Conclusion

We have formulated three different photoluminescent polylactones based NPs, and characterized their physical and chemical properties, protein encapsulation, *in vitro* hemocompatibility, cytocompatibility and particle uptake. Among these formulations, BPLPL-PLGA NPs exhibited stability in physiological conditions, bi-phasic release kinetics, excellent cytocompatibility with no negative influence on cellular functions, optimal uptake characteristics, and hemocompatibility similar to PLGA nanoparticles. Most importantly, BPLPL-based NPs showed intrinsic fluorescence capability inherited from the precursor BPLP. In short, herein we have demonstrated that BPLPL-based NPs are safe, biocompatible material with imaging capability that can be potentially used to fabricate targeted, therapeutic loaded nanocarriers for theranostic applications or utilized as an imaging agent to tag transplanted cells.

## Data Availability Statement

The datasets generated for this study are available on request to the corresponding author.

## Author Contributions

AK, KN, and JY contributed conception and design of the study. AK performed the experiments, data collection, statistical analysis, and wrote the manuscript. NP conducted TEM imaging of nanoparticles whereas DS synthesized the BPLP based polylactones. All authors contributed to manuscript revision, read, and approved the submitted version.

### Conflict of Interest

JY and The Pennsylvania State University have a financial interest in Aleo BME, Inc. and Acuitive Technologies, Inc. These interests have been reviewed by the University's Institutional and Individual Conflict of Interest Committees and are currently being managed by the University. The remaining authors declare that the research was conducted in the absence of any commercial or financial relationships that could be construed as a potential conflict of interest.
